# Field resistance to *Fusarium oxysporum* and *Verticillium dahliae* in transgenic cotton expressing the plant defensin *NaD1*

**DOI:** 10.1093/jxb/eru021

**Published:** 2014-02-06

**Authors:** Yolanda M. Gaspar, James A. McKenna, Bruce S. McGinness, Jillian Hinch, Simon Poon, Angela A. Connelly, Marilyn A. Anderson, Robyn L. Heath

**Affiliations:** ^1^Hexima Limited, School of Botany, The University of Melbourne, Melbourne, Victoria 3010, Australia; ^2^Hexima Limited, La Trobe Institute for Molecular Science, La Trobe University, Bundoora, Victoria 3086, Australia

**Keywords:** Antifungal, defensin, field trials, *Fusarium oxysporum*, transgenic cotton, *Verticillium dahliae*.

## Abstract

The plant defensin NaD1, from *Nicotiana alata*, has potent antifungal activity against a range of filamentous fungi including the two important cotton pathogens, *Fusarium oxysporum* f. sp. *vasinfectum* (Fov) and *Verticillium dahliae*. Transgenic cotton plants expressing *NaD1* were produced and plants from three events were selected for further characterization. Homozygous plants were assessed in greenhouse bioassays for resistance to Fov. One line (D1) was selected for field trial testing over three growing seasons in soils naturally infested with Fov and over two seasons in soils naturally infested with *V. dahliae*. In the field trials with Fov-infested soil, line D1 had 2–3-times the survival rate, a higher tolerance to Fov (higher disease rank), and a 2–4-fold increase in lint yield compared to the non-transgenic Coker control. When transgenic line D1 was planted in *V. dahliae*-infested soil, plants had a higher tolerance to Verticillium wilt and up to a 2-fold increase in lint yield compared to the non-transgenic Coker control. Line D1 did not exhibit any detrimental agronomic features compared to the parent Coker control when plants were grown in non-diseased soil. This study demonstrated that the expression of *NaD1* in transgenic cotton plants can provide substantial resistance to two economically important fungal pathogens.

## Summary

Expression of the plant defensin *NaD1* in transgenic cotton plants increases plant survival, disease tolerance, and yield when grown in soil naturally infested with *Fusarium oxysporum* and *Verticillium dahliae*. Importantly, transgenic plants did not exhibit any detrimental agronomic features.

## Introduction

Fungal diseases are a major threat to global food security (Pennisi, 2010; Fisher *et al.*, 2012) and are the major causal agent affecting agricultural crop yields (Oerke, 2006). Historical methods of pathogen control rely heavily on biological control measures, such as cultivar choice and crop rotation, as well as chemical control measures. Intensive plant breeding and chemical control has allowed farmers to overcome many common plant diseases. However, losses to fungal pathogens still occur in modern production due to the use of susceptible crop species, local environmental factors, and farming practices. The recent use of transgenic plants for the control of insect herbivory and weeds offers another approach to enhance resistance of plants to fungal pathogens.

There are several examples of improved host resistance to fungal pathogens created by the transgenic expression of plant defence molecules. These include proteins from several classes; hevein-like (Koo *et al.*, 2002), glucanases and chitinases (Jach *et al.*, 1995), protease inhibitors (Charity *et al.*, 2005), thionins (Epple *et al.*, 1997; Chan *et al.*, 2005; Oard and Enright, 2006), and defensins (Terras *et al.*, 1995; Gao *et al.*, 2000).

Defensins are widely distributed throughout the plant kingdom and are present in most plant tissues (Lay and Anderson, 2005). They are small, basic proteins with typically four disulphide bonds that confer a highly stable and conserved structure (Thomma *et al.*, 2002). Plant defensins can be divided into two classes based on the presence or absence of a C-terminal pro-peptide (CTPP; Lay and Anderson, 2005).

The plant defensin NaD1, is a well-characterized antifungal protein from the flowers of ornamental tobacco (*Nicotiana alata*). It is a class-II defensin that is produced from a precursor with a 25-amino-acid endoplasmic reticulum signal sequence, a mature defensin domain of 47 amino acids, and a CTPP of 33 amino acids (Lay *et al.*, 2003a,b). NaD1 inhibits the *in vitro* spore germination and growth of several agriculturally important fungal pathogens (Lay *et al.*, 2003a; van der Weerden *et al.*, 2008, 2010).

Plant defensins have a common fold but their sequences are highly divergent and those with antifungal activity may have different mechanisms of action (De Coninck *et al.*, 2013; van der Weerden and Anderson, 2013). Three key events occur during the interaction between NaD1 and the hyphae of *F. oxysporum* f. sp *vasinfectum* (Fov; van der Weerden *et al.*, 2008, 2010). Firstly, NaD1 binds to the cell wall possibly to specific cell-wall proteins. NaD1 then alters the permeability of the hyphal plasma membrane and enters the fungal cytoplasm. Once it enters the cell, NaD1 induces the production of reactive oxygen species and nitrous oxide and the associated oxidative stress accelerates cell death (Hayes *et al.*, 2013). NaD1 adopts a dimeric configuration under physiological conditions that enhances its antifungal activity possibly by enhancing interactions with fungal cell surfaces (Lay *et al.*, 2012).

Fov is the causal agent in Fusarium wilt disease of cotton and is present in almost all cotton-growing regions worldwide (Leslie and Summerell, 2006). It was not until 1993 that an endemic form of this pathogen was identified as an emerging problem to the Australian cotton industry (Kochman, 1995). Like Fov, *V. dahliae* is soil borne, infects the plant vascular system and has a broad host range infecting over 200 plant species (Klosterman *et al.*, 2009). Verticillium wilt has been regarded as an important disease of Australian cotton for many years (Allen, 1995). Initially the management of Fusarium wilt and Verticillium wilt was focused on trash management and crop rotation. However, current farming practices rely on planting the most resistant cultivars available and managing environmental conditions. To date, the use of cultivars with some tolerance to Fov and *V. dahliae* has been the most successful strategy to controlling these diseases (Davis *et al.*, 2006).

NaD1 inhibits the *in vitro* growth of Fov and *V. dahliae* with IC50s of 1.0 μM and 0.75 μM respectively (van der Weerden *et al.*, 2008). The current study reports on the production of transgenic cotton plants expressing *NaD1* under the control of the 35S promoter. Disease resistance against Fov of these plants are examined in greenhouse bioassays. Finally, significant resistance of one transgenic cotton line to the fungal pathogens Fov *and V. dahliae* is demonstrated in 3 years of field trials.

## Materials and methods

### Construction of the pHEX3 binary vector

The coding region of *NaD1* was amplified from the pBS-NaD1 plasmid (Lay *et al.*, 2003a; GenBank accession number AF509566) using primers that introduced a plant Kozak sequence (Joshi, 1987; Lutcke *et al.*, 1987) in front of the start codon. The PCR-amplified product (339bp) was inserted between the cauliflower mosaic virus 35S promoter and terminator sequences in the pFB98/06 vector (gift from Florigene, Australia). The resultant expression cassette was inserted into the pBIN19 binary vector (Bevan, 1984; GenBank accession number U12540) and named pHEX3.

### *Agrobacterium*-mediated transformation of cotton

Transgenic cotton plants were produced by *Agrobacterium*-mediated transformation using the method of Umbec (1991) with some modifications. The binary vector pHEX3 was transferred into competent *Agrobacterium tumefaciens* LBA4404 by electroporation. Cultures of *A. tumefaciens* were then used to infect hypocotyl sections of *Gossypium hirsutum* L. cv. Coker 315. Embryogenic callus was selected on 35mg l^–1^ kanamycin. Following germination, plantlets were transferred to a soil mix and acclimatized in a growth cabinet before transfer to a greenhouse. Primary transformants (T_0_) were self-pollinated and the seed collected.

### Production of homozygous plants

Homozygous lines were identified by their resistance to kanamycin. Approximately 30 segregating T_2_ seed was sterilized and grown on half-strength MS media (Austratec, Australia) containing 10mg l^–1^ kanamycin. T_2_ plants were considered homozygous if all progeny T_3_ plants were resistant to kanamycin and had detectable levels of NaD1 in leaves as determined by enzyme-linked immunosorbent assay (ELISA).

### Adapter ligation-to characterize the T-DNA insertion site

Adapter ligation-(AL) PCR was modified from the method described by Zheng *et al.* (2001). The adapter fragment was prepared using equimolar amounts of AL1 and AL2 (Zheng *et al.*, 2001). An uncloned genomic DNA library was created by the digestion of 1 μg genomic DNA with the six-base restriction endonucleases *Eco*RV, *Stu*I, *Sca*I, and *Sma*I. The fragments of genomic DNA and the adapters were ligated and two rounds of nested PCR were performed. The first round contained the DNA/AL mixture as template and the oligonucleotides ALP1 (Zheng *et al.*, 2001) and RBT1 (5′-AACTCCAGAAACCCGCGGCTGAGTGGCTCC-3′) or LBT1 (5′-ACGGTTTTTCGCCCTTTGACGTTGCAGTCC-3′). The second round used the product of the first round PCR as template with oligonucleotides ALP2 (Zheng *et al.*, 2001) and RBT2 (5′-GGGGTCATAACGTGACTCCCTTAATTCTCC-3′) or LBT2 (5′-AAACAGGATTTTCGCCTGCTGGGGCAAACC-3′). Amplified fragments were separated by gel electrophoresis, gel-purified (MinElute gel extraction kit, Qiagen), and directly sequenced using the second-round oligonucleotides (Macrogen, South Korea).

### Detection of NaD1 by immunoblot analysis

Total soluble proteins were extracted from plant tissue in 50mM H_2_SO_4_ plus 2% (w/v) polyvinylpyrrolidone (PVPP; 1g fresh weight tissue (1.2 ml buffer)^–1^) and insoluble material was removed by centrifugation (18,400 *g*, 10min). The supernatant was collected and protein concentration was determined using the DC protein assay (BioRad). The acid extract (22 μl) was neutralized with 2M TrisHCl, pH 8.5 (5 μl) and added to 7 μl of 4× NuPAGE LDS sample buffer (Invitrogen, Australia) with 5% (v/v) β-mercaptoethanol. Extracted proteins and purified NaD1 from *N. alata* flowers (van der Weerden *et al.*, 2008) were separated by SDS-PAGE on 4–12% (w/v) gradient polyacrylamide gels (Invitrogen) and transferred to a 0.22-μm nitrocellulose membrane (GE Osmonics, USA). Membranes were incubated in 100% isopropanol for 1min at ambient temperature and then blocked in 3% (w/v) BSA in TBS (100mM Tris-HCl pH 7.5, 150mM NaCl) followed by overnight incubation with anti-NaD1 antibody (1/1000 dilution in TBS plus 1% (w/v) BSA; van der Weerden *et al.*, 2008). Membranes were washed (5×10min in TBS with 0.05% (v/v) Tween 20) and then incubated with horseradish peroxidase-conjugated goat anti-rabbit IgG antibody (1:50, 000 dilution in TBS, 1% (v/v) BSA; Pierce). The membranes were washed (5×10min in TBS with 0.05% (v/v) Tween 20), incubated in chemiluminescent substrate (SuperSignal West Pico, Thermo Fisher Scientific, USA) and exposed to autoradiography film (Hyperfilm ECL, GE Healthcare, USA).

### Detection of NaD1 by ELISA

Microtitre plates (96 well Nunc Maxisorp) were coated with 100ng per well of anti-NaD1 antibody (van der Weerden *et al.*, 2008) in 100 μl PBS and incubated overnight at 4 °C. Unbound antibody was removed with wash buffer (0.05% (v/v) PBS and Tween 20), and 3% (w/v) BSA in PBS was added to the wells for 2h at ambient temperature before another wash and addition of antigen. Plant tissue was frozen in liquid nitrogen and ground in a mixer mill (Retsch mn 300) using tungsten balls for 2×10 s at a frequency of 30 s^–1^. The ground tissue was extracted with wash buffer containing 2% (w/v) PVPP (100 μg fresh weight (ml buffer)^–1^) and centrifuged (18,400 *g*, 10min) to pellet insoluble material. Extracts were diluted in wash buffer and incubated in the wells (100 μl) for 1h at ambient temperature. Each sample was assayed in duplicate. Plates were washed and then incubated with 75ng of biotin-labelled anti-NaD1 antibody per well (100 μl) for 1h at ambient temperature. The anti-NaD1 antibody was biotin-labelled using an EZ-Link Sulfo-NHS-LC-Biotinylation Kit (Thermo Fisher Scientific), following the manufacturer’s instructions. Following incubation with the antibody, the wells were washed and horseradish peroxidase-conjugated NeutrAvidin (1:2000 in PBS, Immunopure, Thermo Fisher Scientific) was applied to each well (100 μl) for 1h at ambient temperature. Plates were washed and bound antibody was detected with a peroxidase substrate (ImmunoPure OPD, Thermo Fisher Scientific), following manufacturer’s instructions at 100 μl per well for 3 min at ambient temperature. Colour development was stopped with sulphuric acid (2.5M, 50 μl per well) and the absorbance was measured at 490nm.

### *F. oxysporum*-infected soil bioassay

*F. oxysporum* f.sp. *vasinfectum* 24500 (Australian vegetative compatibility group 01111) was provided by the Department of Primary Industries, Queensland, Australia. The Fov isolate was maintained as glycerol stocks of microconidia and stored at –80 °C.

The glycerol stock (5 μl) was added to 100ml of half-strength potato dextrose broth (12g l^–1^ potato dextrose, Difco) in a 200ml flask and grown for approximately 1 week at 25 °C. The culture (5–10ml) was used to infect approximately 500g autoclaved hulled millet in a 2 L conical flask. The inoculated millet was allowed to stand for 2–3 weeks at ambient temperature before it was incorporated into a pasteurized peat-based soil mix (55.5% peat moss, 18.5% vermiculite, 18.5% perlite, 7.5% sand, 16g l^–1^ Dolomite, 4g l^–1^ Osmocote) at 1% (v/v), by vigorous mixing in a 200-l compost tumbler. The infected soil was transferred to plastic containers (10 l of mix per 13.5 l container). Seed of transgenic line D1 (T3 generation), the parent line Coker 315, a susceptible variety Siokra 1-4, and a less susceptible variety Sicot 189 were sown directly into the containers, 12 seeds per box in a 3×4 array. Sicot 189 and Siokra 1-4 were provided by Dr Steven Allen (CSIRO Narrabri).

Plants were grown for 7 weeks in a greenhouse (daytime 25 °C, overnight minimum 12 °C) and plant survival was measured throughout the bioassay. Disease severity was determined by destructive sampling at the end of the bioassay using a vascular browning index (VBI), where plant stems were sectioned longitudinally and scored. Plants were rated on a scale of 0–5 according to the degree of vascular browning, where 0=no vascular browning, 1=vascular browning restricted to the base of the stem, 2=vascular browning of the hypocotyl, 3=vascular browning of the epicotyl, 4=vascular browning of the complete stem, and 5=plant dead (Lopez-Lavalle *et al.*, 2007). Statistical analysis of the data was conducted by the Statistical Consulting Centre, University of Melbourne. The VBI was analysed using ordinal logistic regression and survival was analysed using logistic regression.

### Field evaluation of transgenic plants

#### *Fusarium* wilt field trials 

The transgenic line D1, the parental line Coker 315, and the less susceptible variety Sicot 189 were grown on a farm in the Darling Downs region of Queensland, Australia during the 2006/07, 2007/08, and 2008/09 cotton-growing seasons. Seed was planted into soil known to be highly infected with Fov. All seed was treated prior to planting with the insecticide Gaucho (Bayer CropScience, Australia) to protect against thrip and aphid damage. For the 2006/07 season, seed was also treated with Mantle (active constituent metalaxyl, Crompton Specialties, Australia) and Terraclor (active constituent quintozene, Crompton Specialties) to control seedling damping off diseases. For the 2007/08 and 2008/09 seasons, half the seed received no fungicide treatment and the other half was coated with the fungicide Dynasty (active constituents azoxystrobin, metalaxyl-M and fludioxonil, Syngenta, Australia) to control seedling damping off diseases. For the 2006/07 season, 800 seeds per plant variety were hand planted in four replicate plots at a density of 10 seeds m^–1^. The D1 homozygous seed (T_5_) had been produced from greenhouse-grown plants. For the 2007/08 and 2008/09 seasons, approximately 2720 seeds per plant variety were mechanically planted in eight replicate plots at a density of 10 seeds m^–1^. The D1 homozygous seed (T_5_) had been produced from field-grown plants. The total number of plants at the start of the trial (initial plant stand) was measured when all viable seeds had germinated. For the 2006/07 trial, the plant stand was measured at 9 d after sowing (DAS). For the 2007/08 trial, the plant stand was measured at 13 DAS, and for the 2008/09 trial, the plant stand was measured at 14 DAS.

At the completion of the trial, plants were assessed for survival, cotton yield, and Fov disease resistance rank (F.rank). Total cotton yield was assessed by hand picking all harvestable bolls and weighing on site. A subset of bolls from each plot was retained for further analysis. Seed was separated from the lint using a laboratory gin and the seed and lint weight was measured. Statistical analysis of the yields (least significant difference analysis of variance) was conducted by the Statistical Consulting Centre at the University of Melbourne or by using the statistical package SPSS version 17. Statistical analysis of surviving plants (Fisher’s exact test) was conducted using the statistical package Minitab16.

The F.rank was determined using the 2008 ranking protocol (http://www.csd.net.au/page/show/21091/) and was calculated after scoring the vascular discoloration in a cross section of the main stem of each plant that had been cut as close as practicable to ground level. A score of 0=no vascular discoloration and 1=less than 5% vascular discoloration. Plants with scores above 1 had vascular discoloration of 5% or more. Plant survival was calculated by dividing the number of plants with a score of 0 or 1 at harvest with the number of plants in the initial plant stand and converting to a percentage. If survival of the test variety (T) was lower than survival of the standard variety Sicot 189 (S) then the equation 100×T/S was used. If T was higher than S then the equation 100+[(T–S)/(100–S)×100] was used. Sicot 189 has an F.rank of 100. A variety with a F.rank of less than 100 is less resistant to Fov than Sicot 189, while a variety with a F.rank of more than 100 is more resistant to Fov than Sicot 189.

#### *Verticillium* wilt field trials 

The transgenic line D1, the parental line Coker 315, and the less susceptible variety Sicala V2 were grown at a farm in New South Wales, Australia during the 2007/08 and 2008/09 cotton-growing seasons. Seed was planted in a field with a history of a high incidence of Verticillium wilt caused by *Verticillium dahliae*. All seed was treated prior to planting with an insecticide (Gaucho, Bayer CropScience Australia) to protect against thrip and aphid damage and some seed was also treated with the fungicide Dynasty to control damping off diseases. For the 2007/08 season, 1000 seeds per plant variety were hand planted in five replicate plots at a density of 10 seeds m^–1^. The initial plant stand was recorded 28 DAS when all viable seed had germinated. For the 2008/09 season, approximately 2720 seeds per plant variety were planted mechanically in eight replicate plots at a density of 10 seeds m^–1^. The D1 homozygous seed (T_5_) was produced from field-grown plants. The plant stand was recorded 34 DAS.

At the completion of the trial, the plants were assessed for cotton yield and Verticillium disease resistance rank (V.rank). Total cotton yield was assessed by hand picking (2007/08) or machine harvesting (2008/09) all harvestable bolls. Yield and statistical analysis were determined as for the Fusarium trials. The V.rank was determined using the 2008 Ranking protocol (http://www.csd.net.au/page/show/21091/). The disease incidence (%) was assessed based on the presence or absence of vascular discoloration in the main stem at or just above ground level at harvest. The % of healthy plants for the test variety (T) or the industry standard Sicala V2 (S) was calculated by subtracting the % disease incidence from 100. The V.rank was then calculated using the same equations as for the F.rank.

#### Disease-free site 

The transgenic line D1 (T_5_ field-grown seed) and the parent line Coker 315 were grown at a farm in the Darling Downs region of Queensland, Australia during the 2008/09 cotton-growing season in a field with no history of cotton diseases. Seed was treated prior to planting with an insecticide (Gaucho) to protect against thrip and aphid damage. Seed was not treated with fungicide. Approximately 4080 seeds per line were machine planted in six replicate plots. At the completion of the trial, plants were assessed for the number of nodes, fruiting nodes, bolls, height, and cotton yield. A subset of line D1 and Coker 315 bolls were retained and the seed was separated from the lint using a laboratory gin. The lint quality was analysed by the Australian Classing Services, Wee Waa, NSW. Seven characteristics were measured: length, uniformity index, short fibre index, strength, micronaire, colour brightness, and colour yellowness.

## Results

### Production and characterization of transgenic cotton plants expressing *NaD1*

Transgenic cotton plants were produced by *Agrobacterium*-mediated transformation of cv. Coker 315 using the binary vector pHEX3 containing *NaD1* under the control of the cauliflower mosaic virus 35S promoter. From one transformation experiment with 395 hypocotyl explants, 21 plants were produced. Ten plants from four events had detectable NaD1 on immunoblots (data not shown). Three plant lines from three separate events (D1, D2, and D3) were selected for further characterization. Plants from the fourth event were not selected as the expression of NaD1 was very low. Primary transgenic plants (T_0_) were allowed to self-pollinate and seed was collected.

Seed from three T_0_ transgenic lines was used for segregation analysis of the *nptII* kanamycin-resistance gene. The percentage of kanamycin-resistant progeny was 79% for line D1, 75% for line D2, and 78% for line D3, which is consistent with the expected Mendelian segregation of 75% for a single T-DNA insertion. Southern analysis of transgenic lines confirmed single-copy events (data not shown). T_1_ plants were grown and homozygous plants identified for further seed production. Lines D2 and D3 were grown to the T_4_ generation and line D1 was grown to the T_6_ generation with no loss of gene expression. When grown in the greenhouse, the three lines did not display any differences in agronomic features such as plant height, fertility, or leaf morphology compared to the Coker 315 parent.

The insertion site of the transgene in line D1 was characterized by AL-PCR. At the site of integration there was a 61 bp deletion of genomic DNA. The 400bp of genomic sequence adjacent to the left border region had no similarity to any known sequence. The 1200bp of genomic DNA directly adjacent to the right border had a 691 bp insert not present in parent Coker 315 DNA. The additional DNA had high identity with mitochondrial sequences from numerous plant species. For example, it shared 96% identity with mitochondrial DNA from *Ricinus communis* (GenBank accession number HQ874649) corresponding to the 5′-untranscribed region and coding region for a protein involved in cytochrome C biogenesis (GenBank accession number ADW96023). The remaining 509bp of genomic sequence adjacent to the right border had no similarity to any known sequence.

**Fig. 1.**
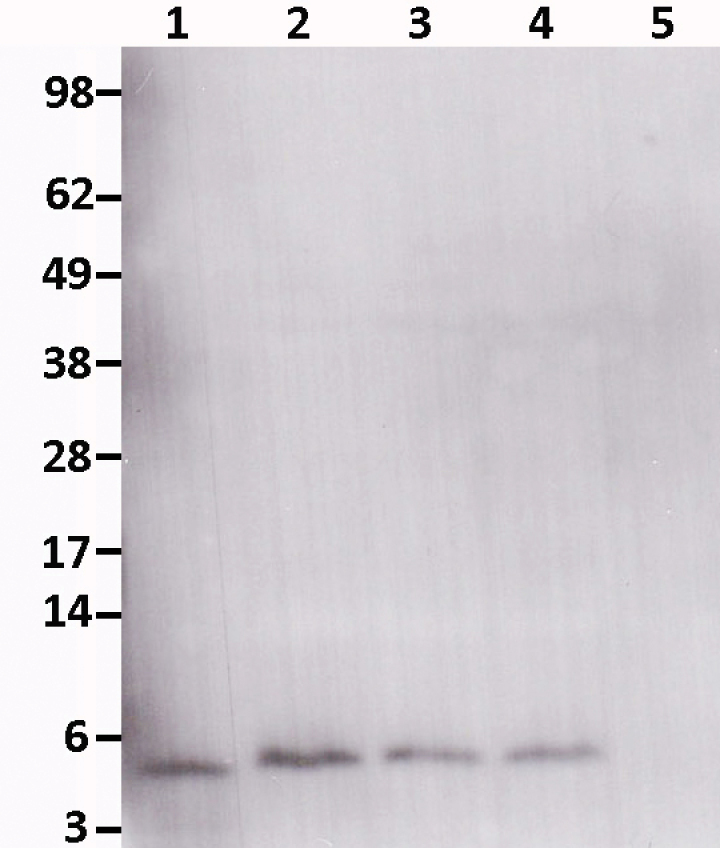
Production of NaD1 in transgenic cotton leaves of 4-week-old homozygous T4 plants. Immunoblot of leaf tissue using anti-NaD1 antibody. Lane 1: 200ng purified NaD1 from *Nicotiana alata* flowers, lane 2: line D2, lane 3: line D3, lane 4: line D1, lane 5: non-transgenic Coker 315.

The three transgenic lines (D1, D2, and D3) produced similar NaD1 levels in leaves of greenhouse-grown plants (Fig. 1). Immunoblot analysis revealed a 5 kDa protein that bound the NaD1-specific antibody and was the expected size for the mature NaD1 protein (Fig. 1). No proteins were detected with the NaD1-specific antibody in the cotton leaf extract of non-transgenic parent line Coker 315. Database searches have not found genes homologous to NaD1 in cotton; class-II defensins are predominantly found in solanaceous species. NaD1 levels in leaf samples from 4- to 6.5-week-old homozygous plants ranged from 0.6 to 10.6 ppm (ng NaD1 (mg wet weight)^–1^; Supplementary Table S1 available at *JXB* online). Line D1 had slightly higher levels of NaD1 compared to lines D2 and D3 when determined by ELISA (Supplementary Table S1).

Field-grown plants of line D1 had NaD1 levels comparable to those in greenhouse-grown plants. For the 2006/07 season, NaD1 levels in 6-week-old plants were around 2 ppm in young leaves, 3–6 ppm in older leaves, 0.3 ppm in stems, and 0.01–0.03 ppm in roots (Supplementary Table S2). Harvested seed contained 1–2 ppm. NaD1 was detected in leaves of plants at all stages of development, from seedlings (3 weeks after sowing) to plants with mature bolls (22 weeks after sowing) (Supplementary Table S3). The highest NaD1 levels were recorded 7–8 weeks after sowing (Supplementary Table S3).

### Greenhouse bioassays with *F. oxysporum*

*NaD1* homozygous plants from the three transgenic cotton lines (D1, D2, and D3) were first assessed in greenhouse bioassays for Fov resistance (data not shown). Line D1 was the only line that exhibited resistance in more than one bioassay and was thus selected for a larger scale bioassay. In this bioassay, survival and Fusarium wilt disease progression in line D1 was compared to three control lines. They were the parental line Coker 315, a susceptible variety Siokra 1-4, and a less susceptible variety Sicot 189.

**Fig. 2.**
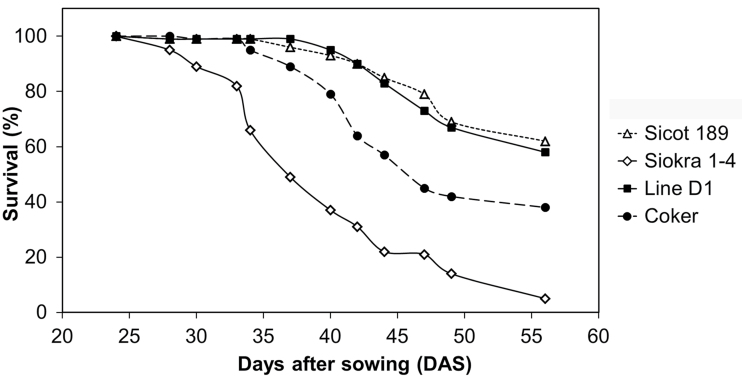
Survival of cotton plants in *Fusarium oxysporum* f. sp. *vasinfectum* (Fov) infected soil in a greenhouse bioassay.

There was a significant difference in plant survival between transgenic line D1 and the parent Coker 315 (*P*<0.01; Table 1 and Fig. 2). The survival of line D1, Coker 315, and Sicot 189 plants was also statistically higher than the susceptible variety Siokra 1-4 (*P*<0.001). After 7 weeks, the susceptible variety Siokra 1-4 had the highest VBI and the less susceptible variety Sicot 189 and the transgenic line D1 had the lowest VBI value (Table 1). There was a significant difference between the VBI of transgenic line D1 and Coker 315 (*P=*0.04). The VBI values of line D1, Coker 315, and Sicot 189 were also statistically significant to that of the susceptible variety Siokra 1-4 (*P*<0.001).

**Table 1.** Survival and disease severity of plants in a greenhouse bioassay in soil infected with *Fusarium oxysporum* f. sp. *vasinfectum* (*Fov*) at 56 d after sowingValues are means. Different superscript letters indicate significant differences (*P*<0.05).

Plant line	No plants germinated	Survival (%)	Vascular browning index
Coker	84	38.1^*a*^	4.0^*a*^
D1	80	57.5^*b*^	3.6^*b*^
Sicot 189	80	61.3^*b*^	3.3^*b*^
Siokra 1-4	87	4.6^*c*^	4.9^*c*^

### Field evaluation of line D1 for *Fusarium* wilt resistance

The performance of line D1 plants against Fov infection was tested under field conditions in the Darling Downs region of Queensland, Australia during the 2006/07, 2007/08, and 2008/09 cotton-growing seasons. Favourable weather conditions (early rain and low temperatures) resulted in high to very high Fov disease incidence, which peaked in the 2007/08 season with severe disease intensity.

**Fig. 3.**
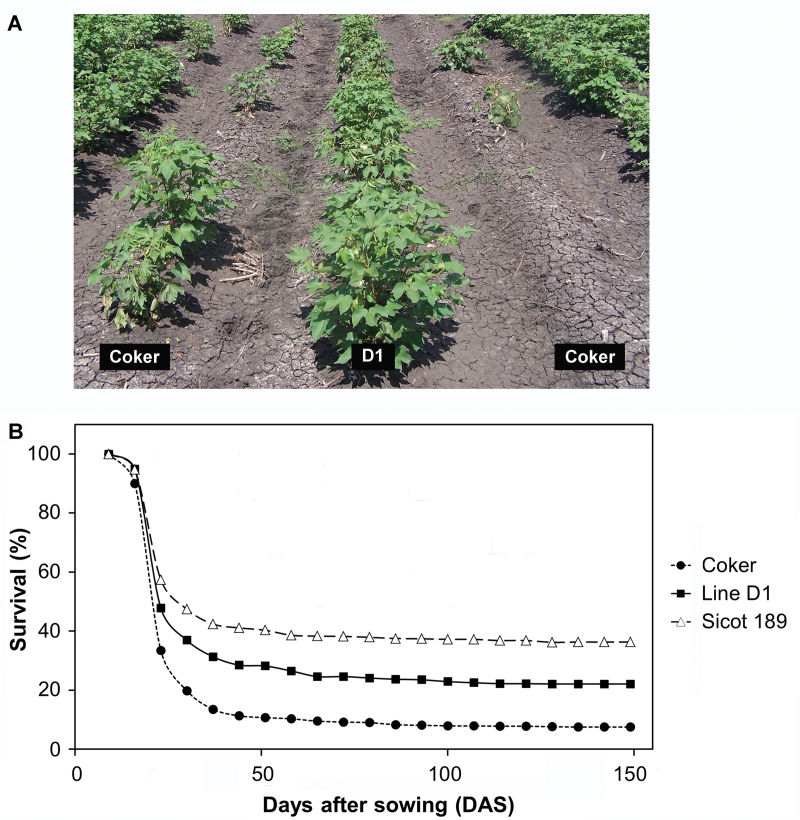
Plants grown during the 2006/07 growing season in a field naturally infested with *Fusarium oxysporum* f. sp. *vasinfectum* (Fov). (A) Line D1, and non-transgenic Coker 315 parent line plants 86 d after sowing. (B) Survival of lines D1, Sicot 189, and Coker 315 during the growing season.

During the 2006/07 field trial Coker 315 exhibited the highest mortality with only 9.1% survival at the end of the season compared with 23.5% and 37.6% survival for the transgenic line D1 and Sicot 189, respectively (Table 2 and Fig. 3). Line D1 also had a higher F.rank compared to Coker 315 confirming the transgenic line was more resistant to Fusarium wilt. There was a statistically higher yield of lint ha^–1^ from line D1 plants compared with Coker 315 (*P=*0.003, Table 2).

**Table 2.** *Fusarium* wilt field trials (2006/07, 2007/08, 2008/09) with transgenic cotton line D1Values are means. The higher the value of the *F*.rank, the higher the disease tolerance. Statistical comparisons were performed within a trial year and not between years. Different superscript letters indicate significant differences (*P*<0.05). Fungicide seed treatment comprised: Mantle (metalaxyl) and Terraclor (quintozene) in 2006/07, and Dynasty (metalaxyl-M, azoxystrobin, fludioxonil) in 2007/08 and 2008/09.

Plant line	Fungicide seed treatment	Survival (%)	F.rank	Lint yield (kg ha^–1^)
2006/07 season				
Coker 315	+	9.1^*a*^	24	169^*a*^
D1	+	23.5^*b*^	66	493^*b*^
Sicot 189	+	37.6^*c*^	100	791^*c*^
2007/08 season				
Coker 315	–	5.9^*a*^	10	76^*a*^
	+	3.7^*b*^	5	62^*a*^
D1	–	13.4^*c*^	15	269^*b*^
	+	8.5^*d*^	14	233^*b*^
Sicot 189	–	20.1^*e*^	100	274^*b*^
	+	21.0^*e*^	100	333^*b*^
2008/09 season				
Coker 315	–	28.9^*a*^	15	516^*a*^
	+	27.1^*a*^	14	542^*a*^
D1	–	49.0^*b*^	66	1260^*b*^
	+	53.2^*c*^	54	1264^*b*^
Sicot 189	–	70.7^*d*^	100	1455^*b,c*^
	+	76.2^*e*^	100	1535^*c*^

Plants in the 2007/08 field trial season had severe Fov disease with very low survival rates and low yields. This was reflected in the low 20% survival of the less susceptible variety Sicot 189. Line D1 continued to exhibit better survival rates and lint yields than Coker 315, with or without fungicide seed treatments (*P*<0.001, Table 2). The F.rank was also higher for line D1 compared with Coker 315. Even though the survival of line D1 was lower than the less susceptible variety Sicot 189, there was no statistical difference in lint yield (Table 2).

Disease pressure for the 2008/09 field trial season was milder; however, line D1 continued to perform better than Coker 315. Line D1 had twice the survival rate and produced 2-fold more lint ha^–1^ compared to Coker 315, with or without fungicide seed treatments (*P*<0.001; Table 2). The F.rank was also up to 4-fold higher for line D1 than Coker 315.

### Field evaluation of line D1 for *Verticillium* wilt resistance

**Fig. 4.**
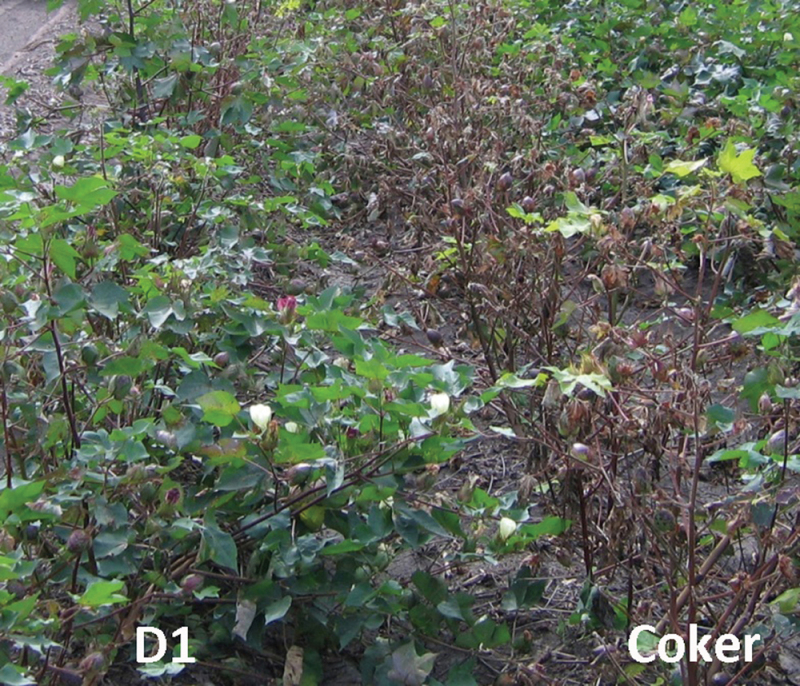
Plants growing in a field naturally infested with *Verticillium dahliae* during the 2007/08 season.

Field trials to assess the performance of line D1 plants against Verticillium wilt were conducted in New South Wales, Australia during the 2007/08 and 2008/09 cotton-growing seasons. Seeds of line D1, parental line Coker 315, and the less susceptible variety Sicala V2 were planted in a field with a history of Verticillium wilt disease. Two seed treatments were compared, one in which the seed was treated with an insecticide for thrip control and one in which the seed was treated with the same insecticide plus fungicide Dynasty for control of seedling damping-off diseases. Disease severity was highest in the 2007/08 season when environmental conditions were most favourable (Fig. 4).

In the 2007/08 Verticillium field trials, line D1 plants produced more lint ha^–1^ than Coker 315, with or without fungicide seed treatment (Table 3). A statistical difference was observed when line D1 (fungicide treated) was compared to Coker 315 line (fungicide treated, *P*<0.001). Line D1 (fungicide treated) had a V.rank of approximately 100 which was comparable to that of the less susceptible variety Sicala V2 (Table 3) and much higher than the V.rank of 10 obtained with Coker 315.

**Table 3.** *Verticillium* wilt field trial (2007/08, 2008/09) with transgenic cotton line D1Values are means. The higher the value of the *V*.rank, the higher the disease tolerance. Statistical comparisons were performed within a trial year and not between years. Different superscript letters indicate significant differences (*P*<0.05). Fungicide seed treatment comprised Dynasty (metalaxyl-M, azoxystrobin, fludioxonil). nt, not tested.

Plant line	Fungicide seed treatment	V.rank	Lint yield (kg ha^–1^)
2007/08 season			
Coker	–	nt	688^*a*^
	+	10	543^*a*^
D1	–	nt	811^*a*^
	+	101	1184^*b*^
Sicala V2	–	nt	nt
	+	100	1828^*c*^
2008/09 season			
Coker	–	73	1615^*a*^
	+	41	1949^*a,b*^
D1	–	103	2083^*b*^
	+	71	1920^*a,b*^
Sicala V2	–	100	1985^*a,b*^
	+	100	2544^*c*^

In the 2008/09 Verticillium trial, line D1 had the highest yield when seed was not treated with fungicide, and there was a statistical difference when compared with untreated Coker 315 (Table 3). As observed in the 2007/08 trial, line D1 (fungicide untreated) had a V.rank very similar to that of the less susceptible variety Sicala V2 and substantially higher than the V.rank obtained with Coker 315.

### Agronomic performance of line D1

The agronomic performance of line D1 in non-diseased soil was assessed during the 2008/09 season at a site not known to harbour cotton fungal pathogens. No visual evidence of fungal disease was observed on the cotton plants during the trial. There were no statistically significant differences between the number of nodes or bolls or plant height or yield for line D1 and Coker 315 (Table 4). There were also no differences in lint characteristics between line D1 and Coker 315 when the length, uniformity index, short fibre index, strength, micronaire, colour brightness, and colour yellowness were analysed (data not shown).

**Table 4.** Agronomic field trial (2008/09) with transgenic cotton line D1Values are means. Independent samples t-test found no statistical significant differences. FP1, fruiting position 1.

Plant line	Number per plant				Height (cm)	Lint yield (kg ha^–1^)
	Total nodes	Fruiting nodes	Total bolls	Bolls in FP1		
Coker	17	12	9	6	86	2133
D1	17	12	10	6	85	2111
*P*-value	0.18	0.91	0.17	0.84	0.63	0.79

## Discussion

Transgenic cotton plants expressing the class-II plant defensin gene *NaD1* were produced by *Agrobacterium*-mediated transformation. Three single-copy transgenic lines from separate transformation events (lines D1, D2, and D3) were selected for further characterization. Line D1 was further characterized under field conditions over three successive years.

NaD1 inhibits the *in vitro* growth of a range of filamentous fungi at concentrations in the order of 0.25–2 μM (Lay *et al.*, 2003a; van der Weerden *et al.*, 2008). In particular, NaD1 has an IC_50_ of 1.0 μM (5.3 ppm) for Fov and 0.75 μM (4.0 ppm) for *V. dahliae in vitro* (van der Weerden *et al.*, 2008). These levels were achieved in the transgenic cotton plants when *NaD1* was expressed under the control of the CaMV 35S promoter. Production of NaD1 in the leaves of the three transgenic cotton lines grown in the greenhouse ranged from 1–9 ppm. Field-grown plants of line D1 produced NaD1 in leaves at levels as high as 6 ppm. The levels of NaD1 are similar to those reported for several class-I plant defensins expressed in transgenic plants (Zhu *et al.*, 1994; Terras *et al.*, 1995; Gao *et al.*, 2000; Portieles *et al.*, 2010). In particular, Gao *et al.* (2000) reported an *in vitro* IC_50_ for alfalfa defensin (alfAFP) inhibition of *V. dahlia* of 5.0 ppm and significant disease resistance in transgenic potatoes expressing alfAFP at concentrations of up to 2.3 ppm (ug (g wet weight)^–1^) in leaves and roots.

This study observed no differences in phenotype between the transgenic cotton lines producing NaD1 and the nontransgenic parent Coker 315 when grown in non-infected soil in the greenhouse or the field, indicating that expression of the *NaD1* transgene did not impact on agronomic performance. There were also no differences in the number and position of nodes and bolls, plant height, and lint yield between transgenic line D1 and Coker 315 when grown in the field at a site not known to harbour cotton fungal pathogens. This is a particularly important finding, as others have reported yield depression from the expression of antifungal transgenes (Zeller *et al.*, 2012). Likewise, others have also reported a fitness cost associated with the maintenance of R-genes (Tian *et al.*, 2003).

NaD1 is a class-II defensin which is produced from a precursor protein with a 33-amino-acid CTPP sequence that targets the defensin to the vacuole for storage (Lay *et al.*, 2003a; Lay *et al.*, 2014). The pHXL3 construct used to produce the transgenic cotton lines has the CTPP sequence intact and subsequent work has shown that NaD1 is deposited in the vacuoles of transgenic line D1 (Lay *et al.*, 2014). Furthermore, phytotoxicity was observed when transgenic cotton plants expressing NaD1 without the CTPP were produced (Lay *et al.*, 2014). In contrast, class-I defensins do not have a CTPP and are secreted into the intercellular spaces (Terras *et al.*, 1995; Gao *et al.*, 2000). Other researchers have reported no obvious phytotoxicity when class-I plant defensins were expressed in tobacco, potato (NmDef02, Portieles *et al.*, 2010), or *Arabidopsis* (Kaur *et al.*, 2012), although potato plants expressing alfAFP produce smaller tubers (Seale and Vordtried, 2010). Kaur *et al.* (2012) have reported that subcellular targeting of a plant defensin to the extracellular space, the vacuole, or the endoplasmic reticulum can have a marked influence on the outcome of a plant–pathogen interaction that varies between pathogens.

### Enhanced resistance to fungal pathogens

Transgenic line D1 had enhanced disease resistance to Fov compared to the non-transgenic Coker 315 parent when grown under field conditions over three successive growing seasons. While all 3 years of the Fusarium wilt trials had similar trends, disease severity varied due to seasonal conditions. Line D1 had between 2-fold and >3-fold higher plant survival when grown in Fov-infected soil when compared to Coker 315 across all three growing seasons, indicating enhanced resistance to Fov. Likewise, the transgenic line D1 also performed better than the non-transgenic Coker 315 control line when grown at a site that was naturally infested with the fungal pathogen *V. dahliae*. The characterization of the line D1 T-DNA integration confirmed that no genes that are likely to be involved in enhanced resistance to fungal disease were interrupted.

The cotton industry regards lint yield as the most important agronomic measurement of cultivar performance. When planted in Fov-infected soil, line D1 had increased lint yields of between 2-fold to >3-fold during the three growing seasons. The improved lint yield in line D1 was largely the result of the improved plant survival, even though line D1 plants had 10% higher yield per plant than Coker 315. When planted in *V. dahliae*-infected soil, line D1 generally had a higher lint yield compared to Coker 315, although this varied by season and fungicide application. The greater improvement in cotton yield observed when assessing resistance against Fov compared to *V. dahliae* may indicate that NaD1 is more effective at controlling Fov. However, Fov is a more aggressive pathogen that causes higher mortality than *V. dahlia* and thus has a greater effect on lint yield.

This enhanced Fov resistance of line D1 reflects results previously reported by others after expression of class-I plant defensins in transgenic plants, although comparison is difficult due to the different pathogens, infection systems, and defensins used. While NaD1 is a class-II defensin and thus targeted to the vacuole, it likely comes into contact with Fov hyphae during their intracellular penetration and rupture of root epidermal and cortex cells en route to the cotton vascular tissues (Hall *et al.*, 2013). The first reported use of a defensin to enhance resistance to a fungal pathogen (*Alternaria longipes)* was in transgenic tobacco expressing the *Rs-AFP2* gene from radish (Terras *et al.*, 1995). Gao *et al.* (2000) reported that transgenic potatoes expressing the alfAFP had significant resistance to *V. dahliae* infection in the greenhouse and the field. Recently, there have been several other reports of class-I plant defensins enhancing fungal resistance in transgenic plants, including papaya (Zhu *et al.*, 2007), rice (Kanzaki *et al.*, 2002; Jha and Chattoo, 2009), potato (Portieles *et al.*, 2010), peanut (Anuradha *et al.*, 2008), wheat (Li *et al.*, 2011), banana (Ghag *et al.*, 2012), and *Arabidopsis* (Kaur *et al.*, 2012). In addition, Dracatos *et al.* (2014) has demonstrated that foliar applications of NaD1 protect oat plants from infection by the crown rust, *Puccinia coronata*.

The field trials described in this study validated the results obtained in the greenhouse bioassay, where the majority of the mortality caused by Fov occurred within 50 d of sowing in both the greenhouse and the field. The development of a laboratory-based bioassay that can successfully identify transgenic lines that perform well under field conditions is very valuable. To date, over 100 publications have reported the transgenic expression of antifungal molecules in plants. However, very few reports relate effectiveness of greenhouse bioassays to field performance or whether the enhanced resistance in the transgenic lines is comparable to that achieved by conventional agricultural practices such as fungicide application. Indeed, only a small number of transgenic lines have been assessed in the field in crops including potatoes (Gao *et al.*, 2000), wheat (Anand *et al.*, 2003), and apples (Krens *et al.*, 2011). The lack of published field trial data may be due to the complexity of translating laboratory results to field situations or the difficulty in negotiating the regulatory requirements required for this type of experiment (Gomez-Galera *et al.*, 2012). Anand and coworkers (2003) described enhanced protection of wheat to *Fusarium graminearum* in the glasshouse, but these lines were not effective under field conditions. In contrast, Gao and coworkers (2000) provided an excellent example of reduced infection in the field although they did not report any impacts on yield from the transgene.

### Future potential

Recently, *F. oxysporum* was voted the fifth most important fungal pathogen in a survey of plant pathologists, based on economic and scientific importance (Dean *et al.*, 2012). With over 120 different special forms that cause disease in most widely grown agricultural crops (with the exception of the grasses), control of *F. oxysporum* by transgenic approaches such as those reported here have the potential to impact many cropping systems (Michielse and Rep, 2009). While NaD1 is effective at inhibiting the *in vitro* growth of *V. dahliae* and Fov, it also inhibits the growth of other commercially imported agricultural pathogens. For example, NaD1 inhibits *Leptosphaeria maculans* and *F. graminearum* (van der Weerden *et al.*, 2010), which are major pathogens of canola and corn, respectively. Both crops are good candidates for the transgenic expression of NaD1 as both can be efficiently transformed and a large percentage of their agricultural production are currently transgenic varieties.

The role played by antimicrobial proteins in plant defence has been well documented (Kaur *et al.*, 2011). Significant effort has been invested in the production of transgenic plant lines with enhanced resistance to fungal disease. However, this effort has not translated into a single commercial plant line possessing enhanced fungal resistance. This is surprising given the potential of this technology to reduce the dependence on chemical pesticides for fungal control or, in the case of Fov and *V. dahliae*, where no effective chemical control method is available.

Line D1, containing *NaD1*, offers considerable yield advantage over the Coker 315 parental line, which is particularly susceptible to Fusarium and Verticillium wilts. It will be interesting to investigate the use of *NaD1* in combination with other antifungal genes to increase the efficacy against Fov and *V. dahliae* and/or broaden the range of pathogens that can be controlled.

## Supplementary material

Supplementary data are available at *JXB* online.

Supplementary Table S1. Levels of expression of NaD1 in leaves of homozygous D1, D2, and D3 transgenic cotton plants grown in the greenhouse.

Supplementary Table S2. Levels of NaD1 in different tissues of homozygous line D1 plants, 6 weeks after sowing in the field during 2006/2007.

Supplementary Table S3. Levels of NaD1 in leaves of homozygous line D1 plants grown in the field during 2006/2007, 3–22 weeks after sowing.

Supplementary Data
