# Supplementary material for: Field resistance to Fusarium oxysporum and Verticillium dahliae in transgenic cotton expressing the plant defensin NaD1
Source: J Exp Bot. 2014 Feb 6;65(6):1541–50. doi: 10.1093/jxb/eru021 (PMC3967090; doi:10.1093/jxb/eru021)
Supplement: Supplementary Data [file supp_65_6_1541__index.html]

Field resistance to Fusarium oxysporum and Verticillium dahliae in transgenic cotton expressing the plant defensin NaD1 — Field resistance to Fusarium oxysporum and Verticillium dahliae in transgenic cotton expressing the plant defensin NaD1 — Supplementary Data 

# Field resistance to *Fusarium oxysporum* and *Verticillium dahliae* in transgenic cotton expressing the plant defensin *NaD1*

## Supplementary Data

Data files

**Files in this Data Supplement:**

- Supplementary Data - Supplementary Data
