# Supplementary material for: Field resistance to Fusarium oxysporum and Verticillium dahliae in transgenic cotton expressing the plant defensin NaD1
Source: J Exp Bot. 2014 Feb 6;65(6):1541–50. doi: 10.1093/jxb/eru021 (PMC3967090; doi:10.1093/jxb/eru021)
Supplement: Supplementary Data [file supp_eru021_jexbot112326_file001.pdf]

Journal of Experimental Botany, Field resistance to *Fusarium oxysporum* and *Verticillium dahliae* in transgenic cotton expressing the plant defensin *NaD1*. Yolanda M. Gaspar, James A. McKenna, Bruce S. McGinness, Jillian Hinch, Simon Poon, Angela A. Connelly, Marilyn A. Anderson, Robyn L. Heath.

Supplementary Table S1 Levels of NaD1 as determined by ELISA in leaves of homozygous D1, D2 and D3 transgenic cotton plants grown in the greenhouse. Plants sampled at 5.5 weeks were a discrete group from the 4 week and 6.5 week group. The first fully expanded leaf was harvested.

**Line**	**Plant No.**	**NaD1 ppm (ng NaD1/mg wet weight tissue)**		
		**4 weeks**	**6.5 weeks**	**5.5 weeks**
D1	1	10.1	1.6	4.5
	2	10.3	2.6	4.2
	3	10.6	3.3	7.0
	4	6.8	3.4	5.7
	**Ave.**	**9.5**	**2.7**	**5.4**
D2	1	8.8	0.6	5.3
	2	7.6	1.2	4.0
	3	9.0	0.6	4.3
	4	8.3	0.8	nt
	**Ave.**	**8.4**	**0.8**	**4.5**
D3	1	9.0	0.7	4.2
	2	7.8	1.8	2.8
	3	8.7	1.8	3.3
	4	9.4	2.6	3.8
	**Ave.**	**8.7**	**1.7**	**3.5**

nt = not tested

Supplementary Table S2 Levels of NaD1 in different tissues of homozygous line D1 plants, 6 weeks after sowing in the field during 2006/2007. NaD1 levels were determined by ELISA.

**Plant**	**NaD1 ppm (ng NaD1/mg wet weight tissue)**			
**No.**	**Roots**	**Stems**	**Young leaves***	**Old leaves#**
1	0.01	0.38	2.0	3.1
2	0.02	0.30	1.7	2.8
3	0.03	0.24	2.1	5.9

*Second fully expanded leaf was harvested

#Third or fourth fully expended leaf was harvested

Supplementary Table S3 Levels of NaD1 in leaves of homozygous line D1 plants grown in the field during 2006/2007, 3 to 22 weeks after sowing. The first fully expanded leaf was harvested. NaD1 levels were determined by ELISA.

**Plant No.**	**NaD1 ppm (ng NaD1/mg wet weight tissue)**								
	**3 wk**	**4 wk**	**5 wk**	**6 wk**	**7 wk**	**8 wk**	**10 wk**	**16 wk**	**22 wk**
1	3.0		1.2		5.8				ns
2	4.4		1.3		8.8				1.8
3	2.9		1.8		5.3				1.5
4		1.0		2.7		8.3	0.8	3.6	
5		2.3		0.9		4.4	1.6	2.2	
6		0.4		4.0		7.5	1.7	nt	

nt = not tested
